# A High-Resolution Proteomic Landscaping of Primary Human Dental Stem Cells: Identification of SHED- and PDLSC-Specific Biomarkers

**DOI:** 10.3390/ijms19010158

**Published:** 2018-01-05

**Authors:** Vasiliki Taraslia, Stefania Lymperi, Vasiliki Pantazopoulou, Athanasios K. Anagnostopoulos, Issidora S. Papassideri, Efthimia K. Basdra, Marianna Bei, Evangelos G. Kontakiotis, George Th. Tsangaris, Dimitrios J. Stravopodis, Ema Anastasiadou

**Affiliations:** 1 Center of Basic Research, Biomedical Research Foundation of the Academy of Athens, 11527 Athens, Greece; tarasliav@hotmail.com (V.T.); stefanialymperi@gmail.com (S.L.); vasopantazo20@hotmail.com (V.P.); 2 Proteomics Research Unit, Center of Basic Research II, Biomedical Research Foundation of the Academy of Athens, 11527 Athens, Greece; atanagnost@bioacademy.gr (A.K.A.); gthtsangaris@bioacademy.gr (G.T.T.); 3 Section of Cell Biology and Biophysics, Department of Biology, National and Kapodistrian University of Athens, Panepistimiopolis, Zografou, 15701 Athens, Greece; ipapasid@biol.uoa.gr (I.S.P.); dstravop@biol.uoa.gr (D.J.S.); 4 Cellular and Molecular Biomechanics Unit, Department of Biological Chemistry, Medical School, National and Kapodistrian University of Athens, Medical School, 11527 Athens, Greece; tegk.84@gmail.com; 5 Center for Engineering in Medicine, Massachusetts General Hospital, Department of Surgery, Harvard Medical School, Boston, MA 02114, USA; MBEI@mgh.harvard.edu; 6 Shriners Burns Hospital, Boston, MA 02114, USA; 7 Department of Endodontics, Dental School, National and Kapodistrian University of Athens, 11527 Athens, Greece; ekontak@dent.uoa.gr

**Keywords:** dental stem cells, PDLSC, SHED, proteomics, cellular functions, molecular pathways

## Abstract

Dental stem cells (DSCs) have emerged as a promising tool for basic research and clinical practice. A variety of adult stem cell (ASC) populations can be isolated from different areas within the dental tissue, which, due to their cellular and molecular characteristics, could give rise to different outcomes when used in potential applications. In this study, we performed a high-throughput molecular comparison of two primary human adult dental stem cell (hADSC) sub-populations: Stem Cells from Human Exfoliated Deciduous Teeth (SHEDs) and Periodontal Ligament Stem Cells (PDLSCs). A detailed proteomic mapping of SHEDs and PDLSCs, via employment of nano-LC tandem-mass spectrometry (MS/MS) revealed 2032 identified proteins in SHEDs and 3235 in PDLSCs. In total, 1516 proteins were expressed in both populations, while 517 were unique for SHEDs and 1721 were exclusively expressed in PDLSCs. Further analysis of the recorded proteins suggested that SHEDs predominantly expressed molecules that are involved in organizing the cytoskeletal network, cellular migration and adhesion, whereas PDLSCs are highly energy-producing cells, vastly expressing proteins that are implicated in various aspects of cell metabolism and proliferation. Applying the Rho-GDI signaling pathway as a paradigm, we propose potential biomarkers for SHEDs and for PDLSCs, reflecting their unique features, properties and engaged molecular pathways.

## 1. Introduction

Tissue engineering represents one of the most promising advances in regenerative medicine, with applications that extend as far as the restoration or regeneration of damaged tissue [1,2,3]. The field aims towards the successful seeding of cells (after first being cultured and/or manipulated in vitro), into a living organism in a manner that allows it to inhabit the environment and restore its proper function. In this context, postnatal stem cells, frequently called adult stem cells (ASCs), or, in the broader term, adult mesenchymal stem cells (MSCs), are fundamental players. They have been isolated from almost all adult tissues of the human body, they display the ability to replenish themselves through self-renewal, and finally they differentiate into specialized cell types and structures upon specific stimuli [4].

A tissue that is abundant with ASCs is the human tooth. It is an excellent source for research studies since it requires minimum invasion for the retrieval of stem cells and, once discarded, it can be used without ethical concerns [5]. Moreover, the fact that tooth development has similarities with other organs of the human body, such as lungs and kidneys [6], has placed human teeth among the most attractive sources of material for ASC research for applications in tissue engineering and medicine.

ASCs are divided into five types of cell populations: Dental Pulp Stem Cells (DPSCs) [7], Stem Cells from Human Exfoliated Deciduous Teeth (SHEDs) [8], Stem Cells from Apical Papilla (SCAPs) [9], Dental Follicle Stem Cells (DFSCs) [10] and Periodontal Ligament Stem Cells (PDLSCs) [11]. These divisions are based on two factors: the area from which they have been isolated and the source.

The stem cells isolated from the oral cavity are of the same embryonic origin, the neural crest [12], and, therefore, it is expected to exhibit similar characteristics and properties. However, as the dental stem cell (DSC) sub-populations are isolated from various regions of the same tissue, they might demonstrate distinct assets that can lead to potential different applications, in both basic research and clinical practice. For example, a scaffold made from collagen has been used with DPSCs to restore human mandible bone defects [13], and therefore are able to form dentin structures [7]. Both SHEDs and PDLSCs have been adopted for a wide range of applications [14]. More specifically, SHEDs can generate a dentin-like odontoblast monolayer [15] and could be used as a source for corneal reconstruction [16]. PDLSCs can form cementum-like structures associated with periodontal ligament-like connective tissue [15], and also have been reported to help creating collagen fibers and mend facial wrinkles in mice [17].

Since characterizing these cells is essential for their potential applications, several studies have been performed, providing either a general characterization of isolated stem cells [15], or comparative analysis of the osteogenic potential of SHEDs and PDLSCs [18], the immunophenotypic and molecular characteristics of the PDLSCs and DPSCs [19,20,21] or SHEDs and DPSCs [22,23,24,25] and the effects of de-cellularized matrices derived from PDLSCs and SHEDs on DPSCs [26]. Moreover, although there are a few studies regarding the proteomic profile of DSCs, they refer to PDLSCs versus DPSCs [27], and SHEDs versus DPSCs [28]. These studies were also performed by using a two-dimensional gel electrophoresis (2-DE) and subsequent peptide identification by matrix-assisted laser desorption/ionization-time of flight mass spectrometry (MALDI-ToF). This approach has various limitations, including a restricted number of obtained proteins and difficulties in isolating acidic, basic and hydrophobic (membrane) proteins [29]. To overcome these limitations, we applied an advanced gel-free nano-LC-MS/MS technology.

In this study, we attempted to molecularly characterize SHEDs and PDLSCs, by identifying the atlas of proteins being expressed in these cells, based on an in-depth proteomic mapping by liquid chromatography-tandem mass spectrometry (LC-MS/MS) analysis. It is the first time, to our knowledge, that such an approach was successfully reported for mesenchymal dental stem cells and has resulted in a large collection of expressed proteins in these distinct stem-cell sub-populations. These results are expected to provide valuable information regarding the molecular characteristics and cellular properties of SHEDs and PDLSCs, to relate specific molecules to these functions, and finally to suggest potential biomarkers associated with their cellular features, properties and prospective applications for their further use and clinical exploitation.

## 2. Results

### 2.1. In-Depth and High-Scale Proteomic Mapping of SHEDs and PDLSCs

SHEDs and PDLSCs, isolated and cultured as previously described [30,31], displayed the typical spindle-shape, fibroblast-like morphology of MSCs, with vigorous microtubule cytoskeleton; SHEDs, in general, demonstrated larger sizes than PDLSCs (Figure S1A). Their mesenchymal nature was indicated by Fluorescence-Activated Cell Sorting (FACS) analysis, employing the triple panel of protein-surface molecules CD73, CD90 and CD105, which by consensus are expressed in MSCs [32]. SHEDs and PDLSCs displayed the above mesenchymal markers at high levels and were negative for CD45 (hematopoietic cell marker), CD34 (hematopoietic stem-cell marker) and CD31 (endothelial cell marker), as expected (Figure S1B). Moreover, they also expressed the stem cell markers [33,34] POU domain, class 5, transcription factor 1 or Octamer-binding protein 4 (Oct-4) and Transcription factor SOX-2 (Sox-2) (Figure S1C). To further confirm the stem cell potent of SHEDs and PDLSCs used in our proteomic assay, their multi-differentiation potential was examined by analyzing their ability to generate osteoblasts, adipocytes and chondrocytes. They were both capable of differentiating into osteo-, adipo- and chondro- specific lineages, following induction, as indicated by the appropriate stains (Figure S2A), and expression of lineage specific markers. *Runt-related transcription factor 2* (*Runx2*), *Collagen a I type I* (*Col1a1*), *osteocalcin* (*BGLAP*) and *alkaline phosphatase* (*ALP*) was found to be upregulated upon stimulation with osteogenic media, as compared to unstimulated ones (Figure S2B). The adipocyte-specific genes, *peroxisome proliferator-activated receptor gamma* (*PPARG*), *CCAAT-enhancer-binding protein alpha* (*CEBPA*), *CCAAT-enhancer-binding protein beta* (*CEBPB*) and *perilipin 1* or *lipid droplet-associated protein 1* (*PLIN1*) were found to be more abundant, following adipogenic differentiation (Figure S2C). A differential upregulation for transcription factors and marker genes for early and late chondroblast differentiation such as the transcription factor *SRY-related HMG-box gene 9* (*Sox9*), *Collagen type II* (*Col2A1*) and *bone morphogenetic protein 4* (*BMP4*) was also observed following chondrocyte induction (Figure S2D).

As the mesenchymal nature of the isolated cells was verified through the above assays, SHEDs and PDLSCs were applied in a gel-free methodology to revel an in-depth and high-scale proteomic mapping by engaging a nano-LC-MS/MS technology. This approach became possible via the LTQ Orbitrap Elite instrumentation with nano-LC engagement, which led to a high-throughput analysis and successful identification of 2032 proteins in SHEDs (Table S1) and 3235 proteins in PDLSCs (Table S2). Comparative evaluation revealed 1516 proteins commonly expressed in both cell types, while 517 were identified only in SHEDs and 1721 were presented exclusively in PDLSCs. By this employment, a deep proteomic profiling of the herein examined dental stem-cell populations was successfully unveiled for the first time.

### 2.2. Sub-Cellular Localization of Commonly Identified Proteins in SHEDs and PDLSCs

**Figure 1:**
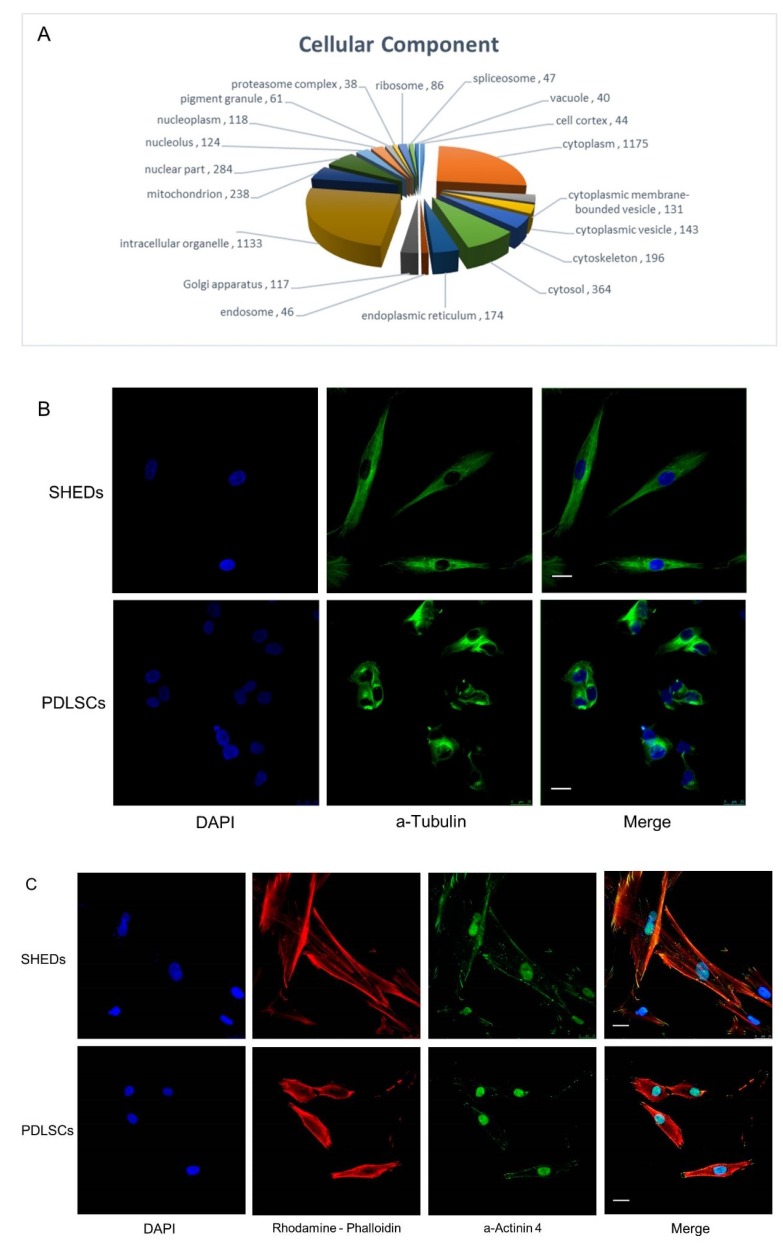

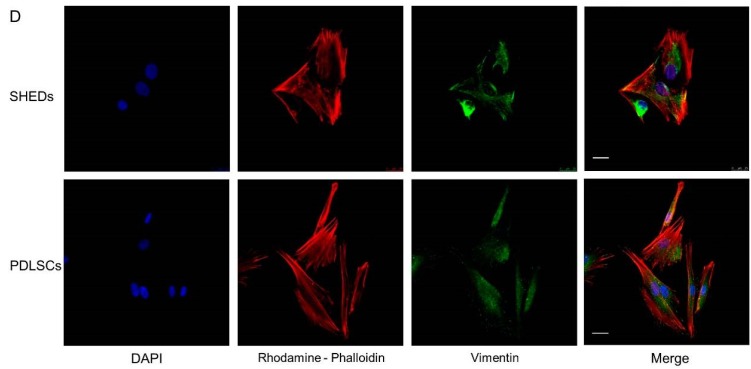
Cellular topology and distribution of the SHED-PDLSC “consensus” proteome generated via nano-LC-MS/MS employment and microscopic visualization of highly abundant cytoskeletal proteins. (**A**) Clustering of the identified, by nano-LC-MS/MS technology, proteins that were expressed in both SHEDs and PDLSCs (“consensus” proteome), into groups based on their cellular topology and distribution (“Cellular Component”). The Gene Ontology (GO) sub-routine of DAVID program was the bioinformatics protocol applied. *p* < 0.05. (**B**–**D**) Representative immunofluorescence images of SHEDs and PDLSCs, captured by confocal microscopy, demonstrating the expression of cytoskeletal proteins. (**B**) a-Tubulin revealed the characteristic spindle-like morphology and filamentous intracellular organization of microtubules cytoskeleton. (**C**) α actinin-4 is found along microfilament bundles and adherent junctions. (**D**) Vimentin is the major cytoskeletal component of mesenchymal cells. Blue: DAPI (nuclear staining). Green: antibodies for tubulin, actinin or vimentin. Red: phalloidin. Magnification: 63×.

Analysis based on sub-cellular localization patterning (*p* < 0.05) of the commonly identified proteins for both SHEDs and PDLSCs (Figure 1A) revealed that most were recognized to reside in the cytoplasm (*n* = 1175) and, more specifically, in intracellular organelles (*n* = 1133). Many molecules were proteins located in the nucleus (*n* = 284), mitochondria (*n* = 238), and participating in ribosomal structure and function (*n* = 86). The significance of protein synthesis and secretion for SHEDs and PDLSCs physiology is indicated by the large number of commonly identified proteins homing the endoplasmic reticulum (ER) (*n* = 174) and Golgi apparatus (*n* = 117), as indicated by the Gene Ontology (GO) sub-routine of DAVID software (*p* < 0.05).

### 2.3. Protein Class-Function of Molecules Identified in Both SHEDs and PDLSCs

Abundant molecules, identified in both SHEDs and PDLSCs by proteomic landscaping, were cytoskeletal proteins, as expected. This was indicated, among others, by the protein coverage, number of unique peptides and mascot score identified (Tables S1 and S2). Several members of tubulin family (α-1B, -1C, and 4A, and β-4B, -3, -2A, and -6), the main component of microtubules, were ranking high in the protein list. These hollow fibers (microtubules) serve as a skeletal system for living cells (Figure 1B) and have the ability to shift through various formations enabling the cell to undergo mitosis or to regulate intracellular transport [35]. Moreover, α actinins (−4 and −1), actin-binding proteins residing along microfilament bundles and adherence-type junctions (Figure 1C), were also in high abundance. Furthermore, vimentin (Figure 1D), a type III intermediate filament that is the major cytoskeletal component of mesenchymal cells [36], was also highly expressed, providing additional evidence for the stemness character of these cells.

**Figure 2:**
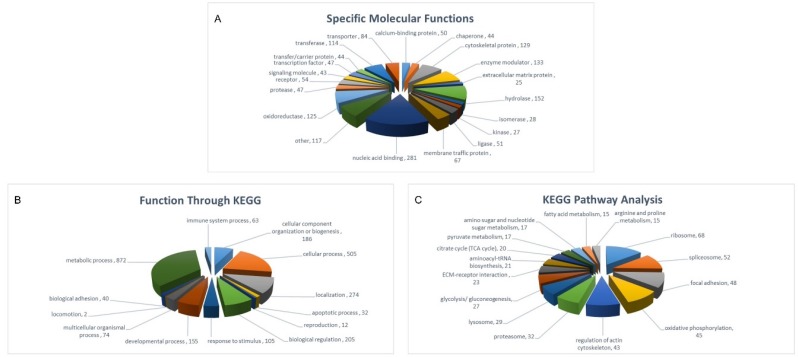
Bioinformatics dissection of the SHED-PDLSC “consensus” proteome generated via nano-LC-MS/MS employment. (**A**) Classification of the SHED-PDLSC “consensus”-proteome contents into several categories of “Specific Molecular Functions”. The Gene Ontology (GO) sub-routine of DAVID program was the bioinformatics tool engaged. *p* < 0.05. (**B**) Categorization of the SHED-PDLSC “consensus”-proteome components into groups carrying broad (general) cellular functions, through KEGG-pathway utilization (“Function Through KEGG”). (**C**) Assembly of the SHED-PDLSC “consensus”-proteome contents into clusters of specific cellular processes, by KEGG-pathway employment (“KEGG Pathway Analysis”).

By performing protein classification of the molecules identified both in SHEDs and PDLSCs, according to their function by the Gene Ontology (GO) sub-routine of DAVID software, the following categories emerged: nucleic acid binding proteins (*n* = 281), hydrolases (*n* = 152), enzyme modulators (*n* = 133), cytoskeletal proteins (*n* = 129), oxidoreductases (*n* = 125), transferases (*n* = 114), transporters (*n* = 84), membrane traffic proteins (*n* = 67), receptors (*n* = 54), ligases (*n* = 51), calcium binding proteins (*n* = 50), proteases (*n* = 47), transcription factors (*n* = 47), chaperones (*n* = 44), transfer/carrier proteins (*n* = 44), signaling molecules (*n* = 43), isomerases (*n* = 28), kinases (*n* = 27), extracellular matrix proteins (*n* = 25), and other classes such as phosphatases, cell adhesion molecules, defenze/immunity proteins, structural proteins, cell junction proteins, surfactants and storage proteins in lower numbers (*p* < 0.05) (Figure 2A).

Through the Kyoto Encyclopedia of Genes and Genomes (KEGG) bioinformatics software, we further clustered the above data into broader categories of function, such as metabolism (*n* = 872), cellular processes (*n* = 505), localization (*n* = 274), biological regulation (*n* = 205), cellular components of organization/biogenesis (*n* = 186), developmental processes (*n* = 155) and responses to stimulus (*n* = 105) (*p* < 0.05). Altogether, these findings indicate the tight regulation of metabolism and biogenesis that adult dental stem cells undergo while remaining in an undifferentiated state (Figure 2B).

Finally, via KEGG-pathway employment, specific cellular processes, in which many of the identified proteins are involved, were reassembled. These include: oxidative phosphorylation (*n* = 45); citrate cycle (TCA cycle) (*n* = 20); glycolysis/gluconeogenesis (*n* = 27); pyruvate metabolism (*n* = 17); amino- and nucleotide-sugar metabolism (*n* = 17); fatty acid metabolism (*n* = 15); ECM-receptor interaction (*n* = 23); aminoacyl-tRNA biosynthesis (*n* = 21); arginine and proline metabolism (*n* = 15); ribosomal (*n* = 68), lysosomal (*n* = 29), proteasomal (*n* = 32) and spliceosomal (*n* = 52) functions; focal adhesion (*n* = 48); and regulation of actin cytoskeleton (*n* = 43) (*p* < 0.05) (Figure 2C). It seems that central metabolism, proteostasis and locomotion constitute indispensable networks for dental stem-cell survival and growth.

### 2.4. Proteins Exclusively Identified in SHEDs

**Figure 3:**
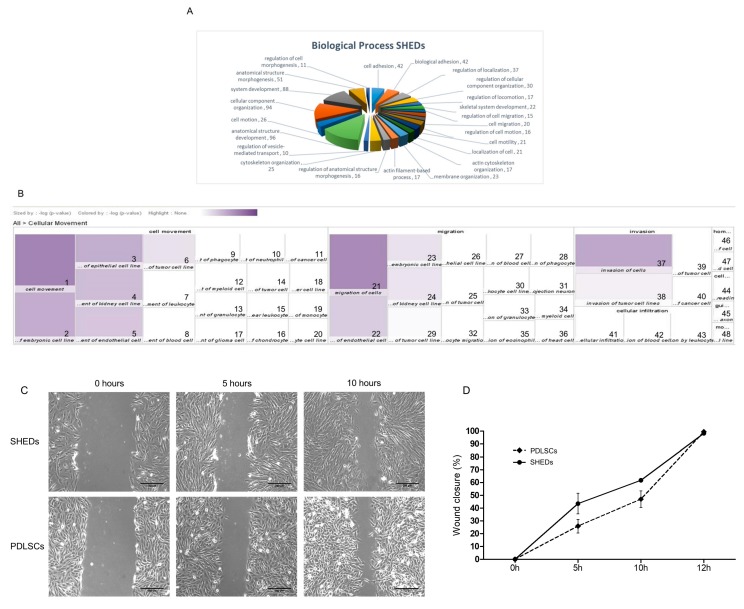

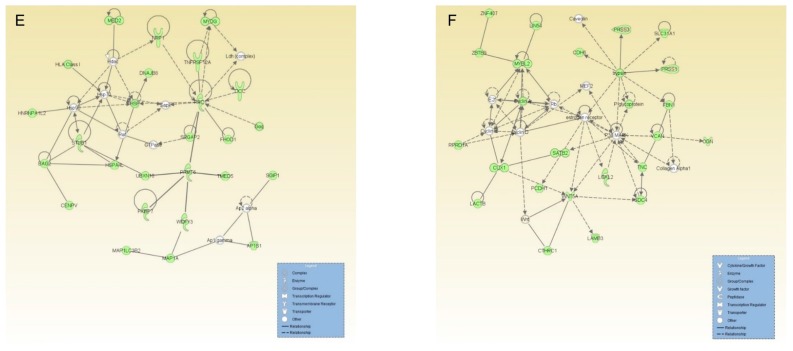
The SHED-specific proteome derived from nano-LC-MS/MS technology utilization. (**A**) Classification of the identified by nano-LC-MS/MS proteins that were exclusively expressed in SHEDs, using their role in general “Biological Processes” as the major criterion for bioinformatics clustering. The Gene Ontology (GO) sub-routine of DAVID software was the bioinformatics protocol applied. *p* < 0.05. (**B**) Heat map of the SHED-specific proteomic list, illustrating 137 proteins involved in cellular movement, migration and invasion. This SHED-specific heat map was created by using IPA. Each legend denotes respective function and *p* value: 1, Cell movement (8.211); 2, Cell movement of embryonic cell lines (6.478); 3, Cell movement of epithelial cell lines (6.418); 4, Cell movement of kidney cell lines (5.962); 5, Cell movement of endothelial cells (5.696); 6, Cell movement of tumor cell lines (5.102); 7, Cell movement of leukocytes (4.451); 8, Cell movement of blood cells (4.388); 9, Cell movement of phagocytes (3.902); 10, Cell movement of neutrophils (3.896); 11, Cell movement of cancer cells (3.757); 12, Cell movement of myeloid cells (3.647); 13, Cell movement of granulocytes (3.249); 14, Cell movement of tumor cells (3.145); 15, Cell movement of mononuclear leukocytes (2.801); 16, Cell movement of chondrocytes (2.784); 17, Cell movement of glioma cells (3.201); 18, Cell movement of macrophage cancer cell lines (2.874); 19, Cell movement of monocytes (2.676); 20, Cell movement of leukocyte cell lines (2.648); 21, Migration of cells (7.910); 22, Migration of endothelial cells (5.535); 23, Migration of embryonic cell lines (5.100); 24, Migration of kidney cell lines (5.067); 25, Migration of tumor cell lines (4.706); 26, Migration of epithelial cell lines (4.242); 27, Migration of blood cells (4.197); 28, Migration of phagocytes (4.149); 29, Migration of tumor cells (4.085); 30, Migration of leukocyte cell lines (3.255); 31, Migration of projection neurons (3.254); 32, Leukocyte migration (3.931); 33, Migration of granulocytes (3.197); 34, Migration of myeloid cells (2.690); 35, Migration of eosinophils (2.774); 36, Migration of heart cells (2.609); 37, Invasion of cells (6.638); 38, Invasion of tumor cell lines (4.865); 39, Invasion of tumor cells (4.447); 40, Invasion of cancer cells (2.759); 41, Cellular infiltration (3.085); 42, Cellular infiltration of blood cells (2.948); 43, Cellular infiltration of leukocytes (2.645); 44, Cell spreading (4.060); 45, Guidance of axons (2.997); 46, Homing of cells (3.348); 47, Homing of blood cells (2.823); 48, Movement of macrophage cancer cell lines (2.829). (**C**) Representative light-microscopy images of time lapses of cell monolayer-scratch wounds, at 0, 5 and 10 h post-scratch. Magnification: 10×. (**D**) Graphical presentation of the wound-healing (closure) percentage (%), at 0, 5, 10 and 12 h, following scratch formation. Data shown are means ± SEM. (**E**) Gaq-, Raf-, STUB1-, RAC1- and PRMT6-dependent molecular network. (**F**) Trypsin-, WNT5A- and Cyclin-A/D/E-dependent protein-interaction network. Protein molecules identified by our analysis are shown in green color, whereas the ones missing from the list (but still belonging to the IPA networks) remain uncolored.

In total, 517 proteins were exclusively identified in SHEDs, appropriately analyzed by applying the Gene Ontology sub-routine of DAVID software and further sub-categorized based on their contribution to the “Biological Process” bioinformatics filter. According to this classification, SHED-specific proteins seem to be implicated in: cell adhesion (*n* = 42), regulation of localization (*n* = 37), cell motility (*n* = 21), regulation of cell migration (*n* = 15), cytoskeleton organization (*n* = 25) and actin cytoskeleton organization (*n* = 17). Moreover, they were characterized as engaged in: anatomical structure development (*n* = 96), system development (*n* = 88), skeletal system development (*n* = 22) and regulation of cell morphogenesis (*n* = 11). The above data indicate that SHEDs express several proteins critically involved in cytoskeletal plasticity, motility, migration, development and morphogenesis, while maintaining their mesenchymal character (Figure 3A).

Moreover, the proteins uniquely identified in SHEDs were suitably processed using the core analysis of Ingenuity Pathway Analysis (IPA) and a heat map was produced (Figure 3B). Interestingly, a concrete reassembly of 137 out of the 517 total SHED-specific proteins were linked to cellular movement, migration and invasion (2.46 × 10^−3^ < *p* < 6.15 × 10^−9^). This is in accordance with the result obtained from wound-healing assays performed for SHEDs and PDLSCs (Figure 3C), demonstrating the strong cellular capacity of SHEDs to migrate towards the artificial wound. SHEDs initially demonstrated a tendency for higher wound-healing activity than PDLSCs, but this difference was statistically insignificant (Figure 3D). In fact, 12 h after the artificial wound was generated, both cell populations revealed 100% wound-healing proficiency, indicating their excellent proliferation and migration capacity to heal a wound.

Additionally, 134 proteins were detected to be implicated in cellular assembly and organization (2.40 × 10^−3^ < *p* < 2.40 × 10^−8^), and 141 ones in cellular function and maintenance (2.41 × 10^−3^ < *p* < 2.40 × 10^−8^). The tight regulation that SHEDs linked to their cellular morphology, assembly and organization is highlighted by the network created via IPA employment, with its core components being identified as the binding partner of Hsp70, STIP1 homology and U-box containing protein 1 (STUB1), Ras-related C3 botulinum toxin substrate 1 (RAC1) and arginine methyltransferase 6 (PRMT6) (Figure 3E). Additionally, in regards to their cellular movement process, the network in Figure 3F illustrated the critical interactions between the core components and their binding partners that were all recognized among proteins exclusively detected in SHEDs (e.g., Gαq and Raf (Figure 3E); trypsin and WNT5A (Figure 3F)). These findings strongly indicate that SHEDs contain an increased number of protein molecules being implicated in the control of cellular plasticity and adaptation to extrinsic and environmental stimuli.

### 2.5. Proteins Exclusively Identified in PDLSCs

**Figure 4:**
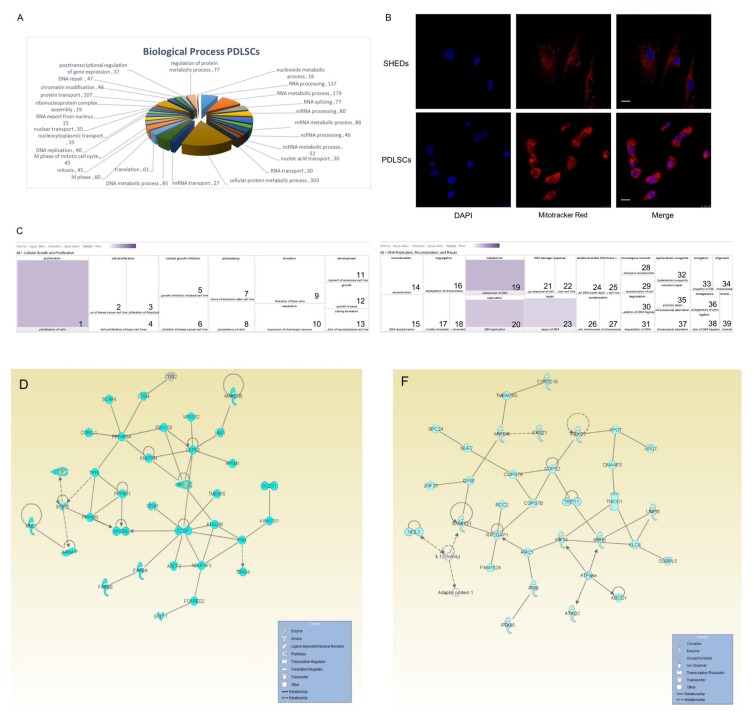

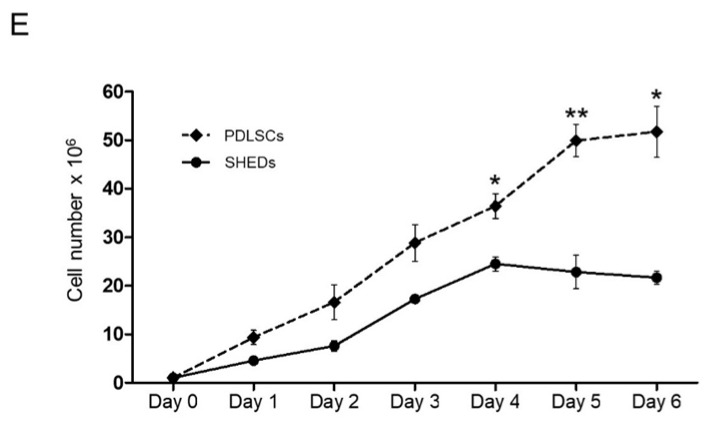
The PDLSC-specific-proteome contents catalogued via engagement of the nano-LC-MS/MS technology. (**A**) Categorization of the identified, via nano-LC-MS/MS advanced protocols, PDLSC-specific proteome into several protein classes, choosing as bioinformatics filter their contribution to general “Biological Processes”. The Gene Ontology (GO) sub-routine of DAVID program was the bioinformatics tool employed. *p* < 0.001. (**B**) Mitochondria visualisation by MitoTracker^®^ Red CMXRos dye. Blue: DAPI (nuclear staining). Magnification: 63×. (**C**) Two heat maps of the PDLSC-specific proteomic catalogue, illustrating 545 proteins involved in cellular growth and proliferation (left panel) and 207 ones engaged in DNA replication, recombination and repair (right panel). These two PDLSC-specific heat maps were generated by employing IPA. Legends indicate respective functions and *p* values: 1, Proliferation of cells (9.539); 2, Cell proliferation of breast cancer cell lines (3.612); 3, Cell proliferation of fibroblasts (3.121); 4, Cell proliferation of tumor cell lines (2.596); 5, Contact growth inhibition of breast cell lines (3.608); 6, Contact growth inhibition of breast cancer cell lines (2.972); 7, Pluripotency of embryonic stem cell lines (3.710); 8, Pluripotency of cells (2.481); 9, Formation of foam cells (4.664); 10, Expansion of cholinergic neurons (2.739); 11, Development of melanoma cell lines (2.739); 12, Growth of yeast (2.664); 13, Colony formation of neuroblastoma cell lines (2.367); 14, Recombination (7.149); 15, DNA recombination (6.826); 16, Segregation of chromosomes (6.827); 17, Segregation of sister chromatids (4.307); 18, Segregation of mitotic sister chromatids (2.722); 19, Metabolism of DNA (9.711); 20, DNA replication (8.840); 21, DNA damage response of cells (5.032); 22, DNA damage response of cervical cancer cell lines (3.543); 23, Repair of DNA (8.406); 24, Double stranded DNA break repair (4.612); 25, Double stranded DNA break repair of cervical cancer cell lines (2.831); 26, Condensation of mitotic chromosomes (3.608); 27, Condensation of chromosomes (3.338); 28, Homologous recombination (2.951); 29, Homologous recombination of cells (2.810); 30, Degradation of DNA fragment (3.044); 31, Degradation of DNA (2.632); 32, Dyskeratosis congenita (4.118); 33, Elongation of DNA (3.475); 34, Alignment of chromosomes (2.972); 35, Excision repair (3.782); 36, Mutagenesis of cells (2.869); 37, Chromosomal aberration (3.643); 38, Ligation of DNA fragment (2.739); 39, Remodeling of chromatin (2.725). (**D**) IPA-mediated assembly of interaction networks for proteins exclusively synthesized in PDLSCs, and critically involved in DNA replication, cell cycle, division, proliferation, DNA recombination and repair. (**E**) Growth curves of SHEDs and PDLSCs. Cells at passage 6 were stained with 0.4% trypan blue solution and standard hematocytometry (Neubauer) was applied to determine cell number and viability. Measurements are from three independent experiments. *: *p* < 0.05. **: *p* < 0.01. (**F**) Ingenuity network was created for PDLSCs regarding cell cycle and cellular assembly and organization. Protein molecules identified by our analysis are shown in blue color, whereas the ones missing from our proteomic catalogue (but still belonging to the IPA molecular networks) remain uncolored.

On the other hand, the majority of proteins exclusively identified in PDLSCs appeared to be mainly classified in the cellular-metabolism network (Figure 4A), as indicated by the Gene Ontology software (*p* < 0.001). Bioinformatics processing, according to the “Biological Process” filter, unveiled a large number of proteins categorized in: cellular protein metabolic process (*n* = 303), regulation of protein metabolic process (*n* = 77), DNA metabolic process (*n* = 85), nucleoside metabolic process (*n* = 16), RNA metabolic process (*n* = 179), mRNA metabolic process (*n* = 86) and non-coding (nc) RNA metabolic process (*n* = 52). Indicative of the intense metabolic activity of PDLSCs is the strong presence of mitochondria observed in these cells when compared to SHEDs, as demonstrated by Mitotracker Red staining (Figure 4B). Additionally, the PDLSC-specific proteins herein recognized, and categorized in Figure 4A, were also clustered in: RNA processing, splicing and transport, as well as, RNA export from nucleus and nucleocytoplasmic transport, translation, chromatin modification and other functions that primarily involve regulatory effects, metabolism and biosynthesis. RNA and protein synthesis, and homeostasis seem to dominate PDLSCs’ intracellular activities.

The 1721 proteins, exclusively identified in PDLSCs, were further analyzed through the employment of IPA bioinformatics tool and two heat maps predominantly emerged (Figure 4C), containing 545 proteins tightly implicated in cellular growth and proliferation (4.29 × 10^−3^ < *p* < 2.89 × 10^−10^), and 207 ones strongly involved in DNA replication, recombination and repair (2.33 × 10^−3^ < *p* < 1.95 × 10^−10^). Interestingly, one of the most concrete networks derived from IPA platform (Figure 4D) contains as core components the centrosomal protein 55 (CEP55), ECSIT signaling integrator, required for proliferation and differentiation of embryonic stem cells [37] and protein phosphatase 4 regulatory subunit 3A (PPP4R3A) which plays a key role in gluconeogenesis of hepatic cells [38]. Moreover, the increased PDLSC cellular division observed in vitro (Figure 4E) can be, at least, partially attributed to the central role of Rac GTPase activating protein 1 (RACGAP1) in the network of Figure 4F, a component of the centralspindlin complex which is located at the central spindle and midbody, and is highly associated with cytokinesis [39]. Overall, PDLSCs revealed a notable mitogenic advantage as indicated by growth curves and the protein molecules identified by our proteomic approach.

### 2.6. Potential Applications in the Biomarkers Field—A Paradigm of Different Markers on the Same Pathway

**Figure 5:**
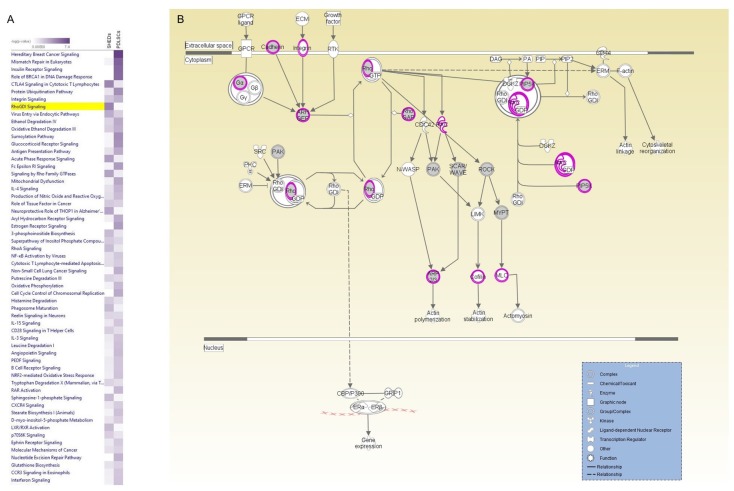
Comparative quantification of molecular pathway’s competence in SHED- and PDLSC-specific proteomes—IPA-mediated reconstruction of Rho-GDI signaling network. (**A**) A unified heat map created via IPA utilization, illustrating selected canonical molecular pathways likely operating, (albeit differently), in both SHED and PDLSC sub-populations. The intensity of purple color is proportional to the -log (*p* value), while the decreased-color intensity denotes the fewer number of genes recognized for each of the indicated functions. (**B**) The Rho-GDI signaling pathway, as generated by IPA application. Annotated proteins from the SHED-specific proteomic list are indicated in purple circle-perimeter, while the ones from PDLSC-specific proteomic catalogue are shown in grey circle-filling.

To comprehend the value of the high-throughput analysis which emerged from the successful landscaping of the proteome of SHEDs and PDLSCs, a direct comparative analysis of the identified proteins was attempted, in several canonical pathways, by suitably engaging the IPA platform. This comparison between SHED and PDLSC expressed proteins, strongly suggested that, even though they share basic molecular functions, they also exhibit differences in distinctive specialized pathways, as illustrated by an integrated heat map, embracing several canonical pathways (Figure 5A).

To validate this scenario, the Rho-GDI signaling pathway (as reconstructed via IPA), was selected since it contains protein molecules critically implicated in various cellular processes, such as cellular migration and reorganization of cytoskeleton (mainly observed in SHEDs) and proliferation (predominantly found in PDLSCs). By annotating the proteins engaged in the Rho-GDI signaling pathway for both SHED (purple circle-perimeter) and PDLSC (grey circle-filling) cell types (Figure 5B), several critical protein molecules proved to be shared between the examined sub-populations, including cadherin, Rho (GDP/GTP) and phosphatidylinositol-4-phosphate 5-kinase (PIP5K). However, SHEDs exclusively express cofilin 2, myosin light polypeptide 9, RAC1, integrin β-2 and integrin α-2 components that are all being related to actin-cytoskeleton integrity, signaling and function, and which could be recognized as potential biomarkers for the successful characterization of SHEDs, related to their distinct nature and properties. On the other hand, PDLSCs expressed exclusively the serine/threonine-protein kinase PAK6, p21-activated protein kinase-interacting protein 1 (PAK1IP1), Rho-associated protein kinase 1 (ROCK1), Wiskott-Aldrich syndrome protein family member 2 (WASF2) and protein phosphatase 1 regulatory subunit 12A (PPP1R12A) that might contribute to the elevated proliferation potency of PDLSCs.

Altogether, it appears that SHEDs and PDLSCs (at least the sub-populations isolated and applied in our assays) have adopted different ways to control their respective actin-cytoskeleton networks, thus dictating the structural and morphological plasticity of dental mesenchymal stem cells during organ’s development and morphogenesis. The abovementioned molecules are suggested, potential biomarkers that are associated with the cells’ features, properties and prospective. These findings need further investigation to concretely identify the molecules that are representative of SHEDs and PDLSCs, and are dynamic biomarkers for different dental sub-populations linked to their cellular roles, functions, and applications. Specific experiments need to be performed, focusing on each one of the abovementioned molecules, to investigate their actual ability to affect structure, adhesion, proliferation, migration and differentiation of SHEDs and PDLSCs. The latter is a proof of the effectiveness of the potency of the cells as well as of their features.

## 3. Discussion

Previous attempts have been made by research groups to catalogue the proteomic content of dental stem cell (DSC) sub-populations. Traditional proteomic approaches were performed, comparing cells being isolated from the same tissue however from different donors with varying health [40] age [28] and different sub-populations of cells [27]. Although 2-DE proteomic strategies provide a first insight to the proteomic landscape of the examined cells, it also exhibits a number of serious limitations, such as the inability to isolate acidic, basic and hydrophobic (membrane) proteins [29], and limited number of obtained proteins. To overcome these limitations, we applied, for the first time, the advanced gel-free nano-LC-MS/MS technology to provide the full proteome of SHEDs and PDLSCs. In total, 2032 proteins were identified in SHEDs and 3235 in PDLSCs. Overall, 1516 were commonly expressed in both cell types, while 517 were exclusively present in SHEDs and 1721 in PDLSCs.

Through thorough examination of the common protein list, several proteins that concurred within the stem-cell nature of SHEDs and PDLSCs were retrieved. As expected, at the top of our list, we re-confirmed the presence of cardinal mesenchymal markers: vimentin, a type III intermediate filament abundantly expressed in mesenchymal tissues [36] as well as an essential regulator of cell division, differentiation and apoptosis that operates in a PKC-dependent phosphorylation manner [41]; galectin-1, an abundant lectin of mesenchymal stem cells (MSCs), which is involved in immunological functions of human mesenchymal stem cells (hMSCs) [42]; and protein DJ-1, which is secreted by adult stem cells (ASCs) and induces their differentiation into osteoblasts [43]. Another important molecule identified in our samples was LASP1, a dynamic focal adhesion protein which is necessary for cell migration and survival in response to growth factors and extracellular matrix proteins, previously reported in a mesenchymal stem cell (MSC) study [44]. Mesenchymal surface markers, such as CD73 and CD105, and potential ones, such as CD29, CD13 and CD44, that were directly engaged in our in vitro experimental protocols and shown to be highly expressed, were also recognized in the nano-LC-MS/MS-mediated large inventory of our obtained proteins. Interestingly, CD166 that was found, by FACS analysis, to follow a stronger expression profile in SHEDs, compared to PDLSCs, that could be identified only in the SHED-protein list and not in the PDLSC one in a statistically significant manner; a finding that needs further investigation.

The anti-apoptotic potential of SHEDs and PDLSCs is highlighted by the presence of 53 proteins involved in the process. Among them, molecules that were identified by nano-LC-MS/MS protocols include annexin A1, heat shock 27 kDa protein 1 and heat shock 70 kDa protein 9 (mortalin). Furthermore, the list is supplemented by annexin A4, annexin A5, cofilin 1, peroxiredoxin isoform 2, 3 and 5, heat shock 60 kDa protein 1 (chaperonin) isoform, heat shock 70 kDa protein 1A and heat shock 70 kDa protein 1B. Of great interest is also the identification of heterogeneous nuclear ribonucleoprotein A2/B1, which controls the G1/S transition of human embryonic stem cells (hESCs) and, therefore, regulates their self-renewal and pluripotency [45], and elongation factor Tu, a protein that was previously proposed as a human embryonic stem cell (hESC) marker [27], which has been herein identified and previously described in PDLSCs [28].

By analyzing the commonly expressed proteins of SHEDs and PDLSCs according to their molecular functions, interesting observations have emerged regarding their metabolic and proliferative capacities. More specifically, out of the 1516 proteins, 872 ones were categorized in the cell-metabolism class, including proteins being implicated in ribosomal, proteasomal and spliceosomal complexes, or other ones involved in oxidative phosphorylation, citrate cycle (TCA cycle), ATP synthesis/binding and pyruvate/fatty-acid metabolism. Moreover, we identified a plethora of enzymes engaged in glycolysis, such as the triosephosphate isomerase 1, aldolase C fructose-bisphosphate, enolases 1 α and 2, glyceraldehyde-3-phosphate dehydrogenase, hexokinases 1 and 2, and lactate dehydrogenases A and B, suggesting that the examined cells might carry the ability to survive and proliferate under hypoxic conditions.

Besides cellular metabolism, the complexity and integrity of cytoskeletal networks in SHED and PDLSC cells are reflected on the 196 identified proteins that belong to the cytoskeleton assembly, including 140 proteins engaged in cell structure and motility. For example, we recognized several myosins (IB, IC, VI, XVIIIA, myosin heavy chain 10 and 9, and myosin light chain 12A and 6), tropomyosins (2B, 3 and 4), tubulins (α 1C and 4A, β 2A and 2C, β 3, and β 5 and 6), actins (1, α 1, and actin related protein 2 and 3), vinculin, and profilin 1 and 2, which are indispensable for biogenesis and organization of cytoskeleton, and are also implicated in differentiation-lineage commitment [28]. An observation of major significance is the identification of cofilin 1, a molecule that regulates actin cytoskeleton and is involved in the control of cell morphology and organization [46], and is abundantly expressed in human mesenchymal stem cells (hMSCs) [47]. Cofilin interacts with destrin or ADF (actin depolymerization factor), which has also been detected in our proteomic catalogue, to depolymerize actin filaments, while it (cofilin) also tethers to other actin-binding proteins to assemble stress fibers and lamellipodia formed by filamentous actin [48,49]. Finally, the identification of 48 proteins participating in focal adhesion highlights the ability of SHEDs and PDLSCs to firmly attach onto surfaces and also modify these adhesions in response to changes in the molecular composition, structure and physical forces exerted by extracellular-matrix environments [50].

The proteins exclusively identified in SHEDs are mainly related to cell migration and motility, morphogenesis and signal transduction. Cell migration is a dynamic process, in which the cytoskeleton undergoes dramatic rearrangements through polymerization and depolymerization of actin, tightly regulated by members of the Rho family of small GTPases [51]. In one of the networks created by IPA, we identified Rac1 as a key player of cellular physiology, thus supporting the suggestion that SHEDs display a higher migratory activity than PDLSCs one. Rac1 is implicated in cell-cell adhesion [52] and mesenchymal-like-driven cell migration [53], while it interacts with FH1/FH2 domain-containing protein 1 (FHOD1), also found to be expressed specifically in SHED cells. Formins act as nucleators of actin filaments and FHOD1 contributes to F-actin organization of mesenchymal cells, as well as to cell migration and invasion [54]. On the other hand, the second network, herein created by IPA, indicates that SHEDs (albeit their strong migration potency) retain the ability to firmly attach onto solid scaffolds. This is also supported by the identification of serine proteinases trypsin 1 and 3, which can degrade proteins of the extracellular matrix, but also by the expression of fibrillin 1, a major component of microfibrils in the extracellular matrix, abundantly synthesized in mesenchymal cells [55]. Additionally, 49 proteins related to cell adhesion could be exclusively recognized in SHEDs, including protein molecules such as collagen XI, XVI and XXIV, contactin 1, dystonin, integrin a2 and b2, OB-cadherin, N-cadherin, K-cadherin and cell adhesion molecule 1.

Interestingly, several proteins implicated in neuronal functions were also included in our SHED-specific proteomic catalogue. Strikingly, molecules such as N-cadherin (cadherin type 1, neuronal), with potential engagement (besides the acquisition of cell’s mesenchymal character) in neuronal processes [56], and neuropilin-1, which participates in axonal fasciculation, neuronal migration, dendritic guidance and repair of the adult nervous system [57], were identified. Moreover, proteins associated with cell migration were detected, including the: (a) FEZ family zinc finger 1, a transcriptional repressor involved in axonal projection and proper termination of olfactory-sensory neurons [58]; (b) apolipoprotein A-I, which (among other functions) induces translocation of cholesterol, phospholipid and caveolin-1 to cytosol in astrocytes [59]; and (c) versican, one of the major extracellular-matrix proteins in the brain, that contains a regulatory domain of neurite growth and synaptic transmission of hippocampal neurons by EGF-R activation [60], which has also been found to interact with fibrillin 1. Tightly associated with morphogenesis, the plexin 1B, a SEMA4D receptor with a critical role in RhoA activation, actin-cytoskeleton structure and plasticity, axon guidance, and invasive growth [61], was included in our list. These are some representative examples of the long catalogue of neuron-associated proteins that characterize our SHED proteome. Their expression could be strongly associated with the neural crest-cell origin of dental pulp, the principal source of SHED-cells isolation [62].

The analysis performed for the proteins exclusively identified in PDLSCs indicated that they carry strong metabolic and proliferative activities. Many PDLSC-specific proteins are related to mitosis and cell cycle. For instance, in one of the networks created by IPA, we can foresee the central role of centrosomal protein of 55 kDa (CEP55), which is essential for centrosome duplication, cell-cycle progression and cytokinesis [63], and of small kinetochore-associated protein (SKAP), whose mouse isoform was previously shown to interact with CEP55 [64] and regulate the transition from metaphase to anaphase [65]. By searching outside of the network context, indispensable protein components of DNA replication and cell division have emerged, therefore strengthening the notion that PDLSCs exhibit higher proliferation capacity, as compared to SHEDs. PDLSDs also seem to proliferate more actively in our experimental in vitro studies, whereas, SHEDs seemed to demonstrate a settled advantage regarding their migratory capacity as compared to PDLSCs.

Remarkably, our PDLSC-specific list contains the: (a) GINS complex subunit 1 (Psf1 homolog), a subunit of GINS complex that plays an essential role in the initiation and progression of DNA replication forks [66]; (b) SPC24, a component of the essential kinetochore-associated NDC80 complex required for chromosome segregation and spindle-checkpoint activity [67]; (c) Cenp-F (or mitosin) needed for kinetochore function and chromosome segregation in mitosis, and kinetochore localization of LIS1, NDE1 and NDEL1 [68]; and (d) pericentrin, a component of the pericentriolar material of centrosome [69] associated with mitotic-spindle organization in mitotic cells [70] and operating as scaffold structure of regulatory elements that support a control mechanism of cell cycle [71]. Additionally, in our protein collection exclusively characterizing the PDLSC cells, we identified the cell cycle-checkpoint protein cyclin B1 and its binding partner cyclin-dependent kinase 1 (CDK1 or CDC2), whose complex results in the formation of maturation promoting factor (MPF), one of the key components regulating cell-cycle progression and especially the G2/M cell-cycle transition [72]. Taken together, all the above data strongly suggest that PDLSCs are characterized by comprehensive cell-cycle control, high proliferation proficiency, and, therefore, rapid growth and expansion.

A comparative study between SHED and PDLSC expressed proteins was performed through the IPA platform, embracing several canonical pathways. The Rho-GDI signaling pathway was selected and further analyzed since it contains protein molecules critically implicated in various cellular processes, such as cellular migration and reorganization of cytoskeleton (mainly observed in SHEDs) and proliferation (predominantly found in PDLSCs). Although, several proteins proved to be shared between the examined sub-populations (cadherin, Rho (GDP/GTP) and PIP5K) others were exclusively expressed in SHEDs and are all being related to actin-cytoskeleton integrity, signaling and function, and which could be recognized as potential biomarkers related to SHEDs distinct nature and properties. SHEDs solely express cofilin 2, myosin light polypeptide 9, RAC1, integrin β-2 and integrin α-2 components, while, on the other hand, PDLSCs express exclusively the serine/threonine-protein kinase PAK6, PAK1IP1, a negative regulator of PAK1 [73], ROCK1, which is required for centrosome positioning and mitotic exit [74], WASF2 and PPP1R12A and might contribute to the elevated proliferation potency of these cells. Therefore, we suggested that these molecules, among others, dictate the structural, morphological and molecular plasticity of the isolated and studied dental mesenchymal stem cells and might represent potential biomarkers associated with the cellular features, properties and prospective of these dental mesenchymal cells.

## 4. Materials and Methods

### 4.1. Proteomics Analysis

#### 4.1.1. LC-MS/MS-Preparation of Samples

Protein samples of ~200 μL, derived from three 75 cm^2^ flasks at passage 8 for SHEDs and at passage 9 for PDLSCs, were washed and precipitated with 600 μL of acetone at room temperature, overnight. Samples were then centrifuged at 3800× *g* for 20 min, and a solution consisting of 8 M urea and 80 mM triethyl ammonium bicarbonate (TEAB) was applied to the pellets. After homogenization, samples were sonicated in a water bath for 30 min, and protein concentration of each sample was estimated with the Bradford assay. For each sample, 200 ng was further used for the experimental protocol applied. Briefly, samples were reduced and alkylated by incubation upon 10 mM dithiothreitol and 55 mM iodoacetamide solutions, respectively, and the digestion was performed by the application of a solution of 1 μg trypsin per 40 μg of protein for 16 h at room temperature, in order to generate peptides. The lyophilization of the peptides occurred in a vacuum concentrator and a solution of 0.1% formic acid in ddΗ_2_O was applied for analysis by liquid chromatography-tandem mass spectrometry (LC-MS/MS). Three independent experiments were carried out for each cell type.

#### 4.1.2. LC-MS/MS and Data Bioinformatics Analysis

The analysis of extracted peptides was performed by employing the bottom-up approach in an LTQ Orbitrap Elite instrument (Thermo Scientific, Rockford, IL, USA). The mass spectrometer (MS) was coupled to a Dionex Ultimate 3000 UPLC system and a C18 Acclaim Pepmap 15 cm column (Thermo Scientific, Rockford, IL, USA), conjugated with an Acclaim Pepmap nano-trap of 2 cm (Thermo Scientific, Rockford, IL, USA), for peptide separation. The phases were: phase A, 99.9% ddH_2_O and 0.1% formic acid, and phase B, 99.9% acetonitrile and 0.1% ddH_2_O. Samples were run at a constant flow rate of 0.3 μL per min, in a linear phase B gradient in 4 h runs, interrupted by 1 h intermediate washing steps of the columns with ddH_2_O. The Orbitrap instrument operated in a positive ion mode and the 20 most intense spectra, as measured at a 60,000 resolution, were chosen for MS/MS fragmentation, employing the higher energy collision dissociation (HCD) function. For HCD of parental ions, the collision energy value was set at 35% and the time of activation was set at 0.1 ms. Ions of *m*/*z* ≥2 were subjected to MS/MS analysis, and the Proteome Discoverer software (Thermo Scientific) and the Sequest search engine (Thermo Scientific) were used to analyze the extracted ion chromatograms (raw files). The database engaged for protein identification searches was the *Homo sapiens* reference proteome, exactly as downloaded from UniProt (UniProt Consortium) (version 2.16) without any further modification [75]. The limits for identification were a precursor-mass tolerance of 10 ppm and a fragment-mass tolerance of 0.05 Da. The cleavage enzyme with a maximum of 0 missed-cleavage parameter was trypsin. A false-discovery rate threshold of 0.5% was set to increase the quality and reliability of all reported protein identifications.

The obtained Universal Protein Knowledgebase (UniProt v2.16; http://www.uniprot.org) accession numbers were processed through the Database for Annotation, Visualization and Integrated Discovery (DAVID) bioinformatics resources v.6.7 (https://david.ncifcrf.gov) [76,77], Kyoto Encyclopedia of Genes and Genomes (KEGG) pathway maps (http://www.genome.jp/kegg) [78,79], Protein ANalysis THrough Evolutionary Relationships (PANTHER) classification system (http://pantherdb.org) [80,81] and Ingenuity Pathway Analysis (IPA; http://www.ingenuity.com) (Qiagen, Redwood City, CA, USA).

### 4.2. Immunostaining and Confocal Microscopy

To visualize cells using confocal microscopy, the examined cells were seeded on cover slips and cultured under standard conditions for 24–48 h at passage 8. They were then fixed in 4% paraformaldehyde (Sigma/Aldrich, St. Louis, MO, USA) solution in 1× PBS working solution for 10 min at room temperature. Cell permeabilization was accomplished with 0.1% Triton-X 100 solution for 10 min, followed by 0.5% Triton-X 100 (Sigma/Aldrich, St. Louis, MO, USA) for 30 min. PDL and SHED primary cells were incubated in 5% BSA blocking solution for 1 h and then in a solution containing the antibody of interest in 0.5% Triton-X 100 and 1% BSA, overnight (~16 h) at 4 °C. Washing three times with 0.5% Triton-X 100 in 1× PBS was followed by cell incubation with the secondary antibody diluted in 0.5% Triton-X 100 in 1× PBS and 1% BSA for 2 h. Next, cells were washed in 0.5% Triton-X 100 before Rhodamine-Phalloidin 1:100 (Biotium, Lab supplies) was added. The cells were washed twice with 1× PBS and VECTASHIELD^®^ Mounting Medium (Vector Laboratories, Inc., 30 Ingold Road, Burlingame, CA 94010 USA) was added on top to prevent rapid photo-bleaching of fluorescence. The primary antibodies used were anti-α Tubulin antibody (ab18251 rabbit 1:1000), anti-α Actinin 4 antibody (ab198608 rabbit 1:100) (Abcam, Cambridge, MA, USA), anti-Vimentin antibody (#3295 rabbit 1:100) (Cell Signaling Technology, Cambridge, MA, USA) and anti-Lamin A/C antibody (ab108595 1:1000) (Abcam). The secondary antibody used was Donkey anti-Rabbit IgG (H + L) Secondary Antibody Fluor^®^ 488 conjugate (Invitrogen, Waltham, MA, USA). Detection of the nucleus was achieved using 4′-6′-Diamidino-2′-phenylindole (DAPI) staining. The samples were analyzed using a Leica TCS SP5 (Leica, Wetzlar, Germany) setup with a 63× objective lens and analyzed using the Leica software, LAS AF.

### 4.3. Migration (Wound-Healing) Assay

For the wound-healing assay, cells at passage 6 were plated at ~80–85% density in 6-well plates (~2 × 10^6^ cells/well) in triplicates and incubated overnight. A 10–200 μL pipette tip was used to scratch a wound through the center of each well, and cells were immediately visualized under a light microscope; this captured image corresponded to time point 0. Cells were then incubated in a 5% CO_2_ humidified environment at 37 °C, until the gap was completely filled with cells. Every 5 h from the time of the scratch, the closure of the generated wound was visualized and appropriate images were taken. The statistical analysis of the percentage closure in the wound-healing assay was performed using the Image-J software.

### 4.4. Growth Curves

Cells at passage 6 were seeded in 6-well plates at a density of ~1.5 × 10^4^ cells per well and collected at 24, 48, 72, 96, 120 and 144 h, using 0.25% trypsin. They were stained with 0.2% trypan blue and counted under a phase-contrast microscope with a hemocytometer. Three independent experiments were performed for each cell sub-population.

### 4.5. Mitochondria Staining

Live cells were stained with MitoTracker^®^ Red CMXRos dye (Cell Signaling Technology) in final concentration of 100 nM for 30 min and fixed with 4% PFA (paraformaldehyde) for 10 min. Then, they were washed 3 times with 1× PBS, followed by the immunofluorescent staining protocol.

### 4.6. Statistical Analysis

We performed statistical analysis by employing the Student’s *t*-test. Significant *p*-value corresponds to *p* < 0.05. Error bars represent standard deviation from the means.

## 5. Conclusions

In this study, we performed, for the first time, a high-throughput molecular comparison of two primary human adult dental stem cell (hADSC) sub-populations, the SHEDs and PDLSCs. A detailed proteomic mapping of SHEDs and PDLSCs, via employment of nano-LC tandem-mass spectrometry (MS/MS), herein reported for the first time, revealing similarities as well as differences regarding the protein expression and the functions that these molecules are likely to be involved. In total, 2032 proteins were identified to be expressed in SHEDs and 3235 in PDLSCs. Overall, 1516 proteins were expressed in both populations, while 517 were unique for SHEDs and 1721 were exclusively expressed in PDLSCs. Further analysis of the recorded proteins suggested that SHEDs are characterized by high cytoskeletal plasticity that renders them excellent candidates for stem-cell-based therapies, during which satisfactory adhesion and migration are required. On the other hand, PDLSCs seem to carry enhanced metabolism and proliferation activities, which could be of great advantage in therapeutic applications whose engrafted cells have to intensively propagate in order to repair and/or replace the damaged tissue(s). Finally, we applied the Rho-GDI signaling pathway as paradigm, and proposed potential biomarkers for SHEDs and for PDLSCs, reflecting their unique features, properties and engaged molecular pathways.

Conclusively, our data are expected to provide a benchmark for future studies regarding the proteomic landscapes, molecular networks and functional responses to extrinsic cues of primary human adult dental stem cells.

## Supplementary Materials

The following are available online at www.mdpi.com/1422-0067/19/1/158/s1.
