# Supplementary material for: A High-Resolution Proteomic Landscaping of Primary Human Dental Stem Cells: Identification of SHED- and PDLSC-Specific Biomarkers
Source: Int J Mol Sci. 2018 Jan 5;19(1):158. doi: 10.3390/ijms19010158 (PMC5796107; doi:10.3390/ijms19010158)
Supplement: Supplementary file 1 [file ijms-19-00158-s001.zip › ijms-252941-Supplementary Material/Supplemetary File.pdf]

Supplementary Information

Human Samples - Cell Cultures

SHEDs and PDLSCs derived from three different donors, aged 9-11 and 11-16 years old respectively, and were isolated by enzymatic methods, as previously described for SHEDs [1] and PDLSCs [2]. Written consent was provided by the parents of the participants donating dental-tissue material. The investigation was carried out following the rules of the Declaration of Helsinki of 1975, revised in 2008. An approval of the Ethics Committee of National an Kapodistrian University of Athens (UoA), Athens, Greece was obtained (#563) according to point 23 of its General Regulation.

Cells were cultured in Dulbecco's Modified Eagle's Medium (DMEM; Gibco, UK), supplemented with 10% heat-inactivated fetal bovine serum (FBS; Biowest, USA), streptomycin/penicillin and L-glutamine, at 37°C in a 5% CO_2 humidified environment. When cultures reached ~90% of confluence (∼5 days), cells were trypsinized and re-plated at a density of ~5.5 x 10^3 cells per cm^2 surface-flask. Cells were frozen at passage 2 and, subsequently, thawed and used for the experimental protocols herein applied.

Flow Cytometry (FACS) Analysis

Cultured cells were trypsinized, rinsed twice with 1x PBS and re-suspended in 1x PBS containing 1% Goat Serum at a density of ~2 x 10^5. They were incubated with specific PE-conjugated antibodies against CD34, CD44, CD73, CD105, CD146 and CD166, and FITC-conjugated antibodies against CD31 and CD90, and APC-conjugated antibodies against CD13 and CD29, for 30 min. The labelled cells were analyzed using the FC-500 Beckman Coulter flow cytometer. PE-IgG1, APC-IgG1 and FITC-IgG1 were used as controls. All antibodies were purchased from BioLegend, USA.

Reverse transcription, semi-quantitative PCR and quantitative PCR

Total RNA from cultured cells at passage 6 was extracted using TRIzol, according to manufacturer's instructions (Invitrogen, USA). The RNA concentration was evaluated utilizing a

NanoDrop 2000 spectrophotometer. cDNA was synthesized with the QuantiTect Reverse Transcription kit, following manufacturer's recommendations (Qiagen, USA). The cDNA was applied for semi-quantitative (semi-q) and quantitative PCR (qPCR). Semi-qPCR reactions were performed using the KAPA Taq PCR Kit (KAPA BIOSYSTEMS) at 95°C for 5 min for the initial denaturation, then 35 cycles of denaturation at 95°C for 30 sec, annealing at 55ºC for 30 sec, extension at 72°C for 60 sec and final extension at 72 °C for 1 min. The primers' sequences are as follows: TACAGCATGTCCTACTCGCAG (forward) and GAGGAAGAGGTAACCACAGGG (reverse) for *Sox2*, GTGTTCAGCCAAAAGACCATCT (forward) and GGCCTGCATGAGGGTTTCT (reverse) for *Oct4* and AGTCAGCCGCATCTTCTTTTG (forward) and CGCCCAATACGACCAAATCC (reverse) for *GAPDH* genes. The amplified PCR products were visualized on a 1.8% agarose gel electrophoresis, stained with ethidium bromide solution and detected under Ultraviolet (UV) light.

For qPCR, SYBR Green PCR Master Mix (KAPA Biosystems, USA) was used, in three independent experiments. Standard cycling conditions were applied: 5 min initial denaturation at 95°C, 40 cycles of 15 sec denaturation at 95°C and 60 sec of combined annealing/extension at 60°C. The real-time primers were designed according to the IDT primer-designing software PrimerQuest on the IDT website (*www.idtdna.com*) and then purchased from the same source. The calculated threshold values were determined by the maximum curvature approach and ΔCt was calculated as Ct_{GAPDH} - Ct_{Sample}. Final values were plotted relative to the value of the control (untreated) cells, which was set to 1x (x: fold). The list of the genes is presented in **Table S3**:

Table S3

Primer sequences of genes amplified by quantitative PCR (qPCR), and used in the evaluation for the three-lineage differentiation competence of SHED and PDLSC sub-populations herein examined.

**Genes**	**Primer sequence (5'-3')**
Runx2	F: GCACCAAGTCCTTTTAATCCA
	R: GCTCAGGTAGGAGGGGTAAGA
COL1A1	F: GAGACCAAGAACTGCCCCG
	R: CGGTGGTTTCTTGGTCGGT

Osteocalcin (BGLAP)	F: GGCGCTACCTGTATCAATGG
	R: TCAGCCAACTCGTCACAGTC
Alkaline phosphatase (ALP)	F: AACACCACCCAGGGGAAC
	R: GGTCACAATGCCCACAGATT
PPARG	F: TCACAAGAACAGATCCAGTGGT
	R: TAAGGTGGAGATGCAGGCTC
CEBPA	F: CTCTTCCCTTACCAGCCGC
	R: GCGATCTGGAACTGCAGGT
CEBPB	F: AGGTCAAGAGCAAGGCCAAG
	R: GGCGATGTTGTTGCGCTC
PLIN1	F: ACTGACAACGTGGTGGACAC
	R: GGTTGTCGATGTCCCGGAAT
Sox9	F: ACTCCTCCTCCGGCATGAG
	R: GCTGCACGTCGGTTTTGG
COL2A1	F: GTTCACGTACACTGCCCTGA
	R: ATGTCAATGATGGGGAGGCG
BMP4	F: CCACAGCACTGGTCTTGAGT
	R: CACTGGTCCCTGGGATGTTC

Cell Differentiation

Approximately 25 x 10^3 cells were seeded per each well of a 24-well plate, for the differentiation assays, whereas ~5 x 10^4 cells were seeded per well of a 12-well plate, for the qRT-PCR assays. Both cell sub-populations used were cultured at passage 6. Cells that did not undergo differentiation were used as controls for the evaluation of differentiation-process proficiency.

Chondrogenic Differentiation

To induce chondrogenesis, cells of ~90% confluency were incubated in a serum-free Chondrogenesis Differentiation Medium (*StemPro* Chondrogenesis Differentiation Kit; Life Technologies, USA) for 14 days. Cells were washed once with 1x PBS and fixed in 4% formaldehyde solution for 30 min, washed once with 1x PBS and then stained in 1% Alcian Blue solution (prepared in 0.1 N HCl) for 30 min. Next, cells were washed three times with 0.1 N HCl and finally ddH_2O was added for acid neutralization. Images from different areas of the examined plates were obtained using a light microscope. Blue staining indicated synthesis of proteoglycans by chondrocytes.

Osteogenic Differentiation

To induce osteogenesis, in cultures displaying ~80–90% confluency, we induced mineralization by incubating cells in an Osteogenesis Differentiation Medium (*StemPro* Osteogenesis Medium; Life Technologies, USA) for 21 days. Then, cells were fixed with 4% formaldehyde for 30 min, rinsed twice with ddH_2O and incubated in a 2% Alizarin Red S solution for 3 min, in order to detect mineralization. Finally, cells were rinsed three times with ddH_2O and images were obtained from different areas of the examined plates using a light microscope.

Adipogenic Differentiation

Adipogenesis was induced with an Adipogenesis Differentiation Medium (*StemPro* Adipogenesis Differentiation Kit; Life Technologies, USA). Cells were grown in the adipose-inducing medium for 14 days and then fixed in 10% formaldehyde for 30 min, rinsed twice with 1x PBS and stained with the Oil Red O working solution (3 parts of 0.3% Oil Red O in isopropanol and 2 parts of ddH_2O) for 10 min. The Oil Red O-positive cells were visualized using a light microscope with the ability to capture snapshots for analysis.

Supplementary Figures

Figure S1 Characterization of primary human dental stem cells (hDSCs): morphological and molecular features

(A). Representative immunofluorescence images captured by engaging confocal-microscopy technology. Cells were stained with phalloidin to picture the cytoskeleton and Lamin A/C for the nuclear envelop marker. Blue: DAPI (nuclear staining). Green: antibodies for Lamin A/C. Red: phalloidin (actin cytoskeleton marker). Magnification: 63x. (B). Flow cytometry was performed for eleven molecules in three independent experiments, and the percentage of positive cells for these protein markers is graphically presented. Statistically significant differences are indicated with asterisks. *: $p < 0.05$. ***: $p < 0.001$. (C). Semi-qPCR was performed using suitable primers for the *Sox2*, *Oct4* and *GAPDH* genes. The amplified PCR products were visualized on an agarose gel electrophoresis, stained with ethidium bromide solution, and a picture was taken under the UV light.

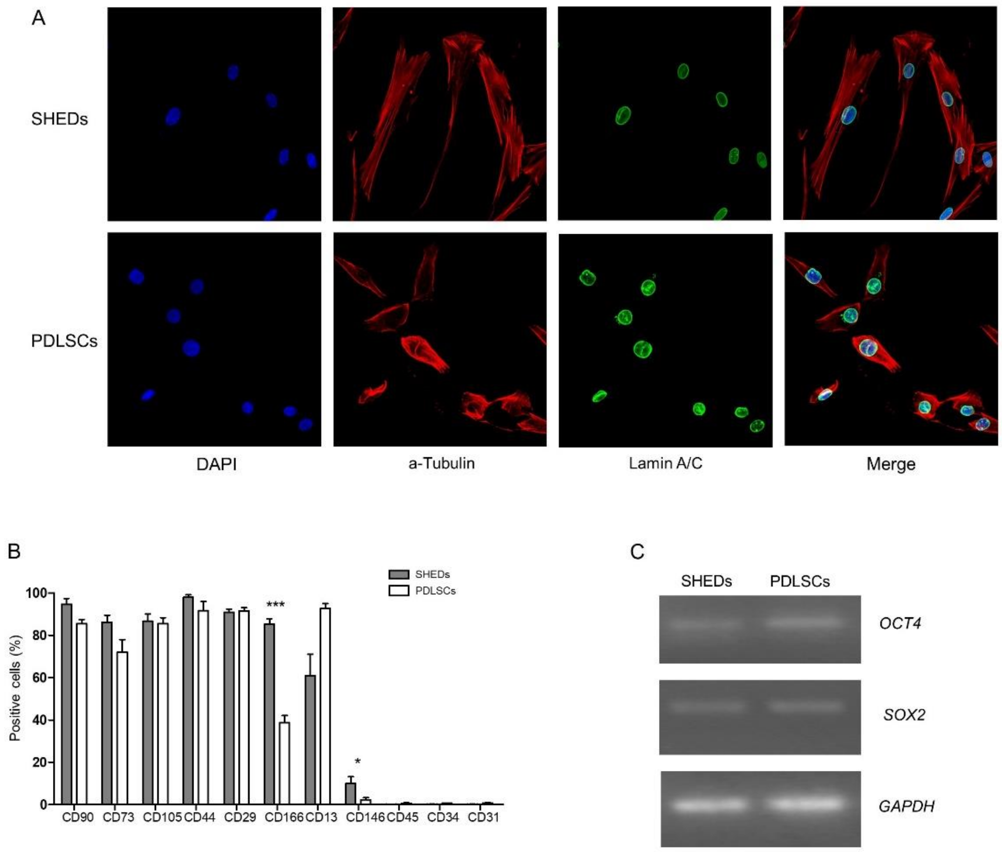

Figure S2 Differentiation proficiency of human dental mesenchymal stem cells (hDMSCs) to the osteogenic, adipogenic and chondrogenic lineages

(A). Three-lineage differentiation assays of SHEDs and PDLSCs. Cells were cultured at passage 6 for 21 days in the appropriate differentiation medium, and then stained with Alizarin Red S for bone-nodule detection (osteogenesis), Oil Red O for reaction with lipid droplets (adipogenesis) and Alcian Blue for chondrocytes (chondrogenesis) identification. Magnification: 10x. (B). The mRNA expression levels of four genes that typify the osteogenic lineage were determined by qRT-PCR in SHEDs and PDLSCs, after they have been grown in the osteogenic medium. (C). The mRNA expression levels of four genes specified for the adipogenic lineage were measured by qRT-PCR in SHEDs and PDLSCs, following their growth in the adipogenic medium. (D). The mRNA expression levels of three genes that characterize the chondrogenic lineage were determined by qRT-PCR in SHEDs and PDLSCs, after they have been exposed to the chondrogenic medium. In (B-D), the results are reported as fold (x) change in gene expression relative to the one of cells cultured in 1x DMEM complete medium, after being normalized to GAPDH values ($\Delta^{\Delta CT}$ method). Columns represent the mean values (± SEM) of three different experiments.

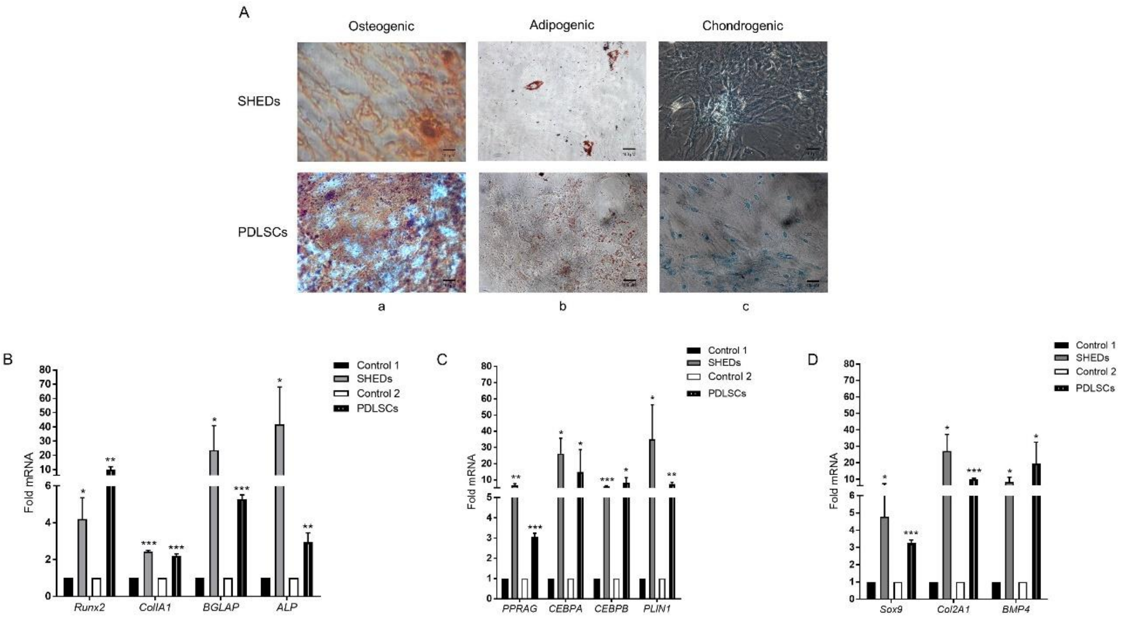

References

1. Miura, M.; Gronthos, S.; Zhao, M.; Lu, B.; Fisher, L.W.; Robey, P.G.; Shi, S. Shed: Stem cells from human exfoliated deciduous teeth. *Proceedings of the National Academy of Sciences of the United States of America* **2003**, *100*, 5807-5812.
2. Seo, B.M.; Miura, M.; Gronthos, S.; Bartold, P.M.; Batouli, S.; Brahim, J.; Young, M.; Robey, P.G.; Wang, C.Y.; Shi, S. Investigation of multipotent postnatal stem cells from human periodontal ligament. *Lancet* **2004**, *364*, 149-155.
